# A trait dataset for freshwater mussels of the United States of America

**DOI:** 10.1038/s41597-023-02635-9

**Published:** 2023-10-27

**Authors:** Garrett W. Hopper, Jamie R. Bucholz, Traci P. DuBose, Kaelyn J. Fogelman, Sean M. Keogh, Megan E. Kubala, Matthew B. Lodato, David H. Nichols, Irene Sánchez González, John M. Pfeiffer, James A. Stoeckel, Jeffrey D. Lozier, Carla L. Atkinson

**Affiliations:** 1 School of Renewable Natural Resources, Louisiana State University and Agricultural Center, Baton Rouge, LA 70803 USA; 2 Department of Biological Sciences, University of Alabama, Tuscaloosa, AL 35487 USA; 3 ORISE Postdoctoral Research Fellow, United States Forest Service, Frankfort, KY 40601 USA; 4 Department of Biological and Environmental Sciences, Troy University, Troy, AL 36082 USA; 5 Field Museum of Natural History, Chicago, IL 60605 USA; 6 National Museum of Natural History, Smithsonian Institution, Washington, D.C. 20560 USA; 7 College of Agriculture, Auburn University, Auburn, AL 36849 USA

**Keywords:** Biodiversity, Zoology

## Abstract

The United States of America has a diverse collection of freshwater mussels comprising 301 species distributed among 59 genera and two families (Margaritiferidae and Unionidae), each having a unique suite of traits. Mussels are among the most imperilled animals and are critical components of their ecosystems, and successful management, conservation and research requires a cohesive and widely accessible data source. Although trait-based analysis for mussels has increased, only a small proportion of traits reflecting mussel diversity in this region has been collated. Decentralized and non-standardized trait information impedes large-scale analysis. Assembling trait data in a synthetic dataset enables comparison across species and lineages and identification of data gaps. We collated data from the primary literature, books, state and federal reports, theses and dissertations, and museum collections into a centralized dataset covering information on taxonomy, morphology, reproductive ecology and life history, fish hosts, habitats, thermal tolerance, geographic distribution, available genetic information, and conservation status. By collating these traits, we aid researchers in assessing variation in mussel traits and modelling ecosystem change.

## Background & Summary

Species traits reflect ecological interactions across broad scales including ontogeny, phenology, and phylogeny and thus when traits are compared across communities, they can be a powerful tool in understanding population dynamics, community ecology, evolutionary biology, and resource management. In the absence of robust occurrence datasets, trait data can serve as an auxiliary source of information because certain traits are often correlated with elevated risks of imperilment. To date, trait-based approaches have been used to study species distribution patterns, abundances, and biotic responses to different types of disturbances^1–3^. Given the escalating biodiversity crisis, a more complete knowledge of species traits is critical to the improvement of natural resource management outcomes, especially for taxa of conservation concern.

One group of concern, the freshwater mussels (Unionoida), are among the most threatened animals globally^4–7^. Specifically in North America, more than 25 extinctions have occurred in the last 100 years and ~65% of remaining species considered endangered, threatened, or vulnerable^8^. Many traits have been used to address contemporary questions regarding the management and conservation of freshwater mussels in the United States of American (Margaritiferidae and Unionidae). For instance, mussel traits have been used to explain species distributions^9–13^, changes in assemblages following habitat alterations^14–17^, potential competition with non-native species^18,19^ responses to climate change^20,21^, local extirpations and extinctions^22,23^, and their functional effects in ecosystems^24–27^. In addition, the distribution and abundance of mussel species is hypothesized to be linked to the distribution and abundance of certain freshwater fishes^9,28^ because nearly all mussel species have larvae that are parasites on fishes. Exceptions include direct development, where larvae bypass the parasitic life-stage and an amphibian serving as a larval host^29^. The relationship with fishes has resulted in a large body of research finding fascinating host attraction and attachment strategies that have evolved among species^30^.

Increasing anthropogenic pressures are changing the structure and function of riverscapes, exacerbating threats to mussel populations and assemblages^31,32^ and elevating the urgency to develop effective tools to identify species responses to environmental change^33^. Many mussel traits were considered critical to the management and conservation of freshwater mussels as early as the 1850s and became the focus of intense study as the pearl button industry began and increased dramatically with creation of the United States of America’s federally funded Fairport Biological Station (Iowa)^34^. The contributions of the Fairport Biological station set the stage for others to address habitat requirements, life history traits, and feeding ecology of mussels^31,35–37^. More recently, conservation agencies such as the United States Fish and Wildlife Service are increasingly incorporating trait data in Species Status Assessments to inform both vulnerability and future adaptability of petitioned species^38^. Given this rich history and increasing wealth of mussel trait and ecological data, a synthesis and standardization of mussel trait data is needed to inform future studies of mussel ecology, management, and conservation.

Here, we accumulated an extensive functional trait, geographic range, and genetic data availability dataset by reviewing accessible literature, databases, and museum collections. Functional traits represent measurable ecological, life-history, morphological, physiological, and behavioural expressions of individuals without reference to environmental context^39^. However, we include information that exists outside of this definition that may inform mussel conservation and management. For example, habitat preferences, such as stream size, velocity, and temperature refer to specific environmental contexts and do not fit this definition and may more accurately be referred to as “attributes”. We use the term “trait” in this dataset to reflect any data type associated with a species in a species X trait data matrix^40^.

We compiled this information into a dataset the expectation that our efforts will (1) guide future large scale projects involving trait-based analysis; (2) act as a foundation that research and conservation projects in the future can build on; (3) and prompt discussion about trait integration and expose knowledge gaps. We acknowledge the dataset is incomplete as mussel traits remain understudied. Our intention is to motivate research on the ecology of this less studied and imperilled taxonomic group. The dataset will be useful to researchers engaged in multispecies, regional, and continental -scale analyses of freshwater mussels of the US that could be expanded more broadly by future efforts. Here, we (1) present the dataset and explain the selection process of data for inclusion; (2) interpret the traits and associated data; (3) define the trait states; (4) explain data formats, storage, retrieval, and limitations. We anticipate the identification of knowledge gaps to fuel future research of these understudied traits.

## Methods

To address gaps in knowledge surrounding mussel traits, we reviewed numerous sources to collate data. A list of species from within the conterminous US (including Alaska) was generated based on the current checklist assembled by the Freshwater Mollusk Conservation Society (FMCS)^41^ and information was sourced primarily from the literature using Google Scholar searches of keywords including trait and scientific names, and by searching existing databases^35,42–45^ Altogether, we consulted >450 peer-reviewed articles, government reports, book chapters, and databases. We often found significant amounts of information for species documented only in state or regional accounts, such as “Freshwater Mussels of…” books or in non-refereed sources such as theses and dissertations, agency web sites or technical reports. Moreover, regional accounts of species cite each other without consistently acknowledging their sources, making it difficult to identify the original source. For each *scientificName* and each *traitName* we have identified a *traitValue* and its associated reference. Most data were extracted from values listed in tables and qualitative descriptions in text. Details relating to data for a particular *traitName* can be found in the description of each *traitName*.The final dataset of traits is represented as a species by traits matrix that includes traits commonly reported in or inferred from species accounts. All references sourced for the dataset generation are included in the SHELDreferencesList2023AUG.xlsx. This file contains the sources for individual entries in the dataset.

### Species in the dataset

Our species list (*scientificName*) follows the recent recommendation of the Names of Freshwater Mollusks Subcommittee of Freshwater Mollusk Conservation Society^41^. Therefore, traits are reported for 301 species of freshwater mussels found in the US (2 families, 7 tribes, 59 genera). Freshwater mussels are here defined as US freshwater mussels from the order Unionoida.

### Shell morphology

Shell size measurements include maximum reported lengths (mm), and mean lengths. When a species mean was reported in multiple studies, we averaged them. Species are considered sculptured if the shell exhibits knobs, pustules, spines, corrugations, or undulations. We classified ((true or false) species sculpturing using photographs from species descriptions in taxonomic keys^42,43,46,47^. We included museum catalogue numbers of photographed shell material when available.

### Reproductive ecology

Fecundity refers to mean number of eggs produced by a single female in one brood. To our knowledge, nostudies strictly evaluated the production of multiple broods per year. Fecundity data were gathered by the original authors by dissecting gravid females during the peak of brooding and counting the number of developing eggs in gills of an individual that contained eggs (i.e., charged gills). Next, we conform to the well-established terminology and refer to two dominant brooding strategies in unionids as long-term species (bradytictic) that continue to brood larvae after they are infectiousand, short-term species (tachytictic) do not^48^. Confusion surrounding brooding strategies in the Margaritiferidae^48^ vrequired us to score all margaritiferid species as “unknown”. We described the marsupium (gills used to brood the larvae) by referring to the outer gills (ectobranchy), or all four (tetrageny). Direct development of larvae has been observed in a few species in the US, but we did not include this trait because it has been paid little attention. Hermaphroditism describes whether hermaphroditic individuals have been observed in populations (true) or not (false).

### Age and growth

Age at maturation refers to the earliest report in years of viable gametes in the species. We coded this trait as 0 if reproductively mature individuals were reported within their first year of life. Longevity refers to the life span in years. Growth rates are reported as a separate spreadsheet and include all values available from the literature (SHELDgrowthRates2023AUG.csv). In most cases, Von Bertanlanffy’s K was used to estimate growth rates (K) and does not represent true growth rates. Values of K are dimensionless parameter that relates how quickly the growth curve becomes asymptotic. Because methods for calculating growth rates can vary, it may be necessary to view the original sources when making intra- and inter-specific comparisons.

### Larval morphology

Larvae description includesmeasurements, shapes, and possession of hook-like structures based on Hoggarth (1999)^49^. The assignments are mutually exclusive, and each trait is discrete. The original accounts were determined by removing larvae from the marsupia of preserved female unionids in museum collections^49^. Larvae representing 30 genera were described while viewed under a scanning electron microscope (SEM). In the event that SEM images of larvae were available for a species, but a shape was not defined in the article or report we matched the SEM image to previously published illustrations^46,49^. Definitions of the recorded shape follow those of early efforts^49^. Other larval traits included in the dataset are maximum length and maximum height (µm).

### Host data and host infection strategy

We used the Freshwater Mussel Host Database (https://mollusk.inhs.illinois.edu/57-2/; accessed January-December 2020) to collate available data on fish host use (primary or secondary) either from laboratory or natural transformations for all unionid species^44^. It was necessary to supplement with references from the literature because not all mussel species are represented in the Freshwater Mussel Host Database. We included only fish species that are recognized by the American Fisheries Society (AFS)^50^ and thus limit the dataset to 247 fish species tested as hosts for at least one unionid mussel species. Next, we created a fish species by mussel species association matrix with 1 indicating the fish species served as a host and 0 indicating the fish is not known to serve as a host and summed each mussel species column. We followed a similar procedure for fish genera and families to count the number of host genera and families, respectively, reported to serve as larval hosts. The matrix can be found in SHELDfishMusselMatrix2023AUG.csv. Finally, we categorized the primary host infection strategy used for each mussel species following previously established classifications^34^ These include broadcasting, female sacrifice, conglutinate, and mantle lure strategies.

### Thermal tolerance and upper lethal limits

We used data compiled during a systematic literature review of lethal thermal tolerance upper lethal limits (ULL) for unionid mussels in US faunal region^45^. All thermal tolerance data is categorized by life stage and species and when available includes measurements of lethal temperature where 5% of the population will experience mortality (LT05), median lethal temperature where 50% of the population will experience mortality (LT50), and critical thermal maximum (CTMax). When one measurement of a test type of ULL has been reported in the literature for a single species and life stage, that value is reported as the mean. If multiple estimates of ULL were found in the literature for a test type, species, and life stage combination, we reported the minimum, maximum, and the mean ULLs for that species in the dataset.

All thermal tolerance data must be interpreted with caution and consideration of the test conditions. Details of confidence intervals, acclimation temperature, test duration, or CTMax rate of change surrounding each of the ULL measurements reported in this dataset can be traced to the original references and a review of thermal tolerances among freshwater mussels^45^.

### Habitat, stream characteristics, and geographic distribution

This section consists of trait sub-categories describing species associations with substrate type, lentic and lotic systems collated from the literature. Twenty-five habitat associations are included in this section, including seven substrate classes. In some cases, habitats are described based on similarities to habitats where shell material or freshly dead individuals were collected. Particle size classification are provided to facilitate conversion to numerical values^51^. Pfeiffer *et al*.^52^ recently used digitized natural history collections data to estimate various occurrence-derived species attributes; 18 of those attributes are included here.

We also estimated mussel biogeography by recording species presence/absence in recognized freshwater mussel faunal zones^53^. Specifically, we created shapefiles for each faunal zone by aggregation of Hydrological Unit Code (HUC) at the 10-digit level (HUC10’s)in the *sf* R Package^54^ and used georeferenced occurrences to estimate faunal region occupation in the *CoordinateCleaner* R package^55^. We considered a species present in a region if it had >2 occurrences, questionable if 1–2 occurrences, and absent if no occurrences. See https://github.com/seanmkeogh/SHELD_biogeo for R script and associated shapefiles.

### Conservation status

We reported conservation status of species (i.e., endangered, threatened, or petitioned/candidate reported on the US Federal Register) searchedSeptember^56^, 2022) U.S. Fish and Wildlife Services Species Data Explorer^56^. We also included NatureServe’s global (range-wide) conservation status ranks (G- ranks; https://explorer.natureserve.org/; searched January 1-October 1, 2022). NatureServe conservation status ranks are a valuable complement to legal status designations assigned by government agencies such as the US Fish and Wildlife Service and the National Marine Fisheries Service in administering the US Endangered Species Act (ESA).

### Genetics

To investigate the distribution of genetic data availability and identify data gaps across US freshwater mussels, we collated information on genetic information at the species-level. We focused on the availability of traditional low-throughput: DNA sequence data from regions of the mitochondrial genome, the nuclear genome, multilocus microsatellite genotyping data, and high-throughput sequencing data. To gather information about available mitochondrial and nuclear DNA sequence data for each species we searched the GENBANK sequence database from the National Center for Biotechnology Information (www.ncbi.nlm.nih.gov/genbank/). The principal approach for data discovery was to search the taxonomy database within GENBANK for the genus + species names (including known taxonomic synonyms as needed) and scoring species with data as “YES” if mitochondrial or nuclear sequences were available. If additional data types were present beyond single gene sequences for a species (whole mitochondrial genome, expressed sequence tags, RNA/transcriptome sequences, whole genome) in GENBANK or REFSEQ databases these were also noted. Because no centralized databases exist for microsatellite genotyping data, the availability of microsatellites for each species was collated using literature searches performed using Google Scholar (scholar.google.com). Search terms included “genus” AND “microsatellites”. Studies with microsatellite primer discovery (in the absence of population specific diversity and differentiation estimates) were also included. DOI information for publications was also recorded.

To aggregate information on the availability of high throughput sequencing data, we searched the National Center for Biotechnology Information Sequence Read Archive (SRA) for each species (https://www.ncbi.nlm.nih.gov/sra, searched between October 5–10, 2022). For SRA data, values for each species include “NO” if no data were detected, “WGS” if whole genome shotgun sequencing was available “RNA” if RNA sequencing or transcriptome data was available, “RAD” if Restriction Site Associated DNA sequencing (any method) was available, “AHE” if Anchored Hybrid Enrichment or other hybrid capture data was available, and “Microbiome16S” if 16S amplicon sequencing of the bacterial community was available. Multiple values are separated by colons.

## Data Records

The dataset is available at Figshare^40^.

### First file

Identity**:** SHELDspeciesTraitMatrix2023AUG.xlsx

**Size:**215 KB

**Format and storage mode:** xlsx

**Contents:** This file contains the raw data with all *traitValues* and is considered the final dataset

**Variable information:**

**scientificNameAuthority**: Scientific name used under the Linnaean classification system with taxonomic authority of the scientific name and date published^41,57,58^

**scientificName:** Scientific name used under the Linnaean classification system. The contents of this column can be used as a key between the spreadsheets included in this dataset.

**family:** Family recognized by FMCS founded on Turgeon (1998), updated by Williams *et al*. (2017) and then updated during FMCS 2021 as recommend by the Names of Freshwater Mollusk Committee.

**subFamily**: Subfamily identification recognized by FMCS founded on Turgeon (1998), updated by Williams *et al*. (2017) and then updated as of FMCS 2021 as recommend by the Names of Freshwater Mollusk Committee.

**tribe:** Tribe identification per Pfeiffer *et al*. (2019). Name recognized by FMCS founded on Turgeon (1998), updated by Williams *et al*. (2017) and then updated as of FMCS 2021 as recommend by the Names of Freshwater Mollusk Committee.

**genus:** Generic name recognized by FMCS founded on Turgeon (1998), updated by Williams *et al*. (2017) and then updated as of FMCS 2021 as recommend by the Names of Freshwater Mollusk Committee. These refer to those used by the Linnaean classification system.

**species:** Specific epithet recognized by FMCS founded on Turgeon (1998), updated by Williams *et al*. (2017) and then as of FMCS 2021 as recommend by the Names of Freshwater Mollusk Committee. These refer to those used by the Linnaean classification system. These names were used as search terms in some cases during the data retrieval phase.

**commonName:** Common names recognized by FMCS founded on Williams *et al*. (2017) and updated as of FMCS 2021 as recommend by the Names of Freshwater Mollusk Committee. These names were used as search terms in some cases during the data retrieval phase.

**tsn:** Taxonomic Serial Number from Integrated Taxonomic Information System. Represent a persistent identifier to facilitate tracing name changes in the future. May not reflect all name changes accepted by FMCS 2021. NA- no data of this classification available for the species.

**natureServeElement:** Unique identifier for NatureServe queries.

**meanLength:** Mean of shell lengths. Measured across the anterior-posterior plane of the shell. NA- no data of this classification available for the species.

**maxLength**: Maximum shell length (length description) in millimeters. Some records may estimate asymptotic length. We assumed that the difference between maximum observed length and asymptotic length is negligible. Measured across the anterior-posterior plane of the shell. NA- no data of this classification available for the species.

**shellSculpture:** Species are considered sculptured (true) if the shell exhibits knobs, pustules, spines, corrugations, or undulations. Mussels without the aforementioned sculpturing were scored “false” in the dataset This binary classification (true or false) was based on photographed material found on MolluscaBase, Musselp^43^, taxonomic keys, and species descriptions^46,47,59^.

**larvalDescription:**

Descriptions that reflect two discrete larval characteristics. The term “hooked” is applied indiscriminately to independently derived structures that function similarly, to grab onto the host fish. Hookless refers to the absence of such a structure. Here, the larvae are hooked or hookless combined with terms describing their shape based on Hoggarth^60^.

hookless_ligulate: Without hook. “Axe-head shape”, much higher than long. Dorsal margin short and straight. Anterior and posterior margins are parallel to about 80% from dorsal to ventral becoming evenly curved to point of maximum lateral inflation distant from the dorsal margin. Ventral margin only slightly curved.

hookless_subligulate: Without hook. Much higher than long. Dorsal margin slightly curved. Anterior and posterior margins straight to slightly incurved dorsally. Margins become straight again at about 40% from dorsal margin, but slightly divergent. Maximum lateral inflation about 80% from dorsal margin.

hookless_subspatulate: Without hook. Dorsal margin straight. Lateral margins straight, divergent dorsally, ventrally parallel; ventral margin gently curved.

hookless_subelliptical: Without hook. Dorsal margin straight. Lateral margins gently curved but unequal. Maximum inflation of posterior margin at about 70% from dorsal to ventral. Maximum inflation of the anterior margin at about 40% from dorsal margin. Ventral margin is narrowly rounded.

hookless_elongate_oval: Without hook. Dorsal margin slightly curved outward. Lateral and ventral valve margins more or less round in outline, with maximum inflation of both side margins at about 50% from dorsal to ventral.

hookless_subrotund: Without hook. Dorsal margin slightly curved outward. Lateral and ventral valve margins not quite round in outline. Maximum inflation of side margins about 50% from dorsal to ventral.

hookless_subtriangular: Without hook. Dorsal margin is straight; posterior margin gently and evenly curved; anterior margin curved broadly to is maximum inflation at ~70% from dorsal to ventral. Ventral terminus is broadly pointed.

hookless_fabelliform: Without hook. “bean-shaped”, much longer than high having a straight dorsal margin, narrowly rounded anterior and posterior margins, broadly curved ventral margin. Anterior and posterior margins equal, with their points of maximum inflation at about 30% from dorsal margin.

hooked_ligulate: Possessing a hook. “Ax-head shape”, much higher than long. Dorsal margin short and straight. Anterior and posterior margins are parallel to about 80% from dorsal to ventral becoming evenly curved to point of maximum lateral inflation distant from the dorsal margin. Ventral margin only slightly curved.

hooked_subelliptical: Possessing a hook. Dorsal margin straight. Lateral margins gently curved but unequal. Maximum inflation of posterior margin at about 70% from dorsal to ventral. Maximum inflation of the anterior margin at about 40% from dorsal margin. Ventral margin is narrowly rounded.

hooked_subtriangular: Possessing a hook. Dorsal margin is straight; posterior margin gently and evenly curved; anterior margin curved broadly to is maximum inflation at ~70% from dorsal to ventral. Ventral terminus is broadly pointed.

hooked_pyriform: Possessing a hook. Dorsal margin is straight. Posterior margin broadly curving, with maximum inflation at about 30% from dorsal to ventral. Anterior margin more broadly curved than posterior margin. Maximum anterior inflation at about 40% from dorsal margin. Lateral margins slightly incurved ventrally, producing a narrowly rounded ventral terminus located 40% from posterior to anterior.

hooked_depressed_pyriform: Possessing a hook. Posterior margin is broadly curved; anterior margin is rounded. Lateral margins meet at a narrowly rounded, nipple-like ventral terminus located about 50% from posterior to anterior.

NA: no data of this classification available for the species.

**larvalHeight:** Mean height of larvae in micrometers (µm) measured perpendicular to the hinge. Max height is given if no mean is reported. NA- no data of this classification available for the species.

**larvalLength:** Mean length of larvae in micrometers (µm) measured parallel to the hinge. NA- no data of this classification available for the species.

**matureAge:** Mean, median, or modal age at maturity in years for females. Where different ages at maturity were gathered for distinct populations, the best supported records were averaged. Species that are known to mature prior to age 1 are defined as 0. Male maturity age was accepted where female data were not available. NA- no data of this classification available for the species.

**maxAge:** Longevity in years based on life in the wild wherever available. Where not indicated, the record was assumed to be from the wild. If wild records were not known, records for captive individuals were considered. NA- no data of this classification available for the species.

**growthRate:** Binary indicator (true or false) of the availability of growth rate data for each species. Literature sources often evaluated growth rates inconsistently. We dealt with this by including a separate sheet of growth rates, SHELDgrowthRates2023AUG.csv. That sheet details growth rates, sample sizes, location of the study, and the data source.

**fecundity**: Refers to mean number of eggs produced by a single female in one brood. There were not studies strictly evaluating the production of multiple clutches per year. Fecundity data were gathered by the original authors by dissecting gravid females during the peak of brooding and counting the number of developing eggs in all charged gills of an individual. NA- no data of this classification available for the species.

**brood:** long_term indicates long (bradytictic)- or short_term indicates short term (tachytictic) brooding strategy; long term brooders hold larvae from fall (September to December) to spring (March to June) or summer (June to September), whereas short term brooders hold larvae from winter to spring or summer. NA- no data of this classification available for the species.

**marsupialGills:** Indicates that brooding of larvae occurs in outer gills only (ectobrancy) or all four gills (tetrageny). This trait is a distinct characteristic of some genera. NA- no data of this classification available for the species.

**hermaphrodite:** Indicates reports of hermaphroditic populations (true). If no hermaphroditism has been reported the species is assumed to have male and female sexes (false). If this trait has not been explicitly reported or investigated, we report as NA.

**hostInfectionStrategy:** Category of primary host infection strategy. For example, some species display mantle lures, but also release secondary conglutinates for a time. Categories are defined as:

broadcast-Broadcasting involves larvae release whereby host fish are encountered by chance; no apparent adaptations to attract hosts; Passive entanglement and release of free larvae are not separated.

sacrifice-Species that specialize on molluscivorous host fish; high fecundity, small body sizes, high male:female ratios, and high larvae infection of molluscivorous fishes support this; May also broadcast into sediment to increase chance of infection via suction feeding by host fish.

conglutinate-Release larvae in discrete clusters, that can resemble fish prey items that facilitate host infection; Can be further categorized by functional attraction of specialists. 1) Pelagic,2) Demersal, 3) Mucoid, 4.) superconglutinate.

mantle_lure-Lures displayed by gravid females that mimic a variety of fish prey items; Can be further categorized large (Tribe:Lampsilini) and cryptic lures (Tribe: Lampsilini) or mantle magazines (Tribe: Quadrulini).

kinetic_lure-The conglutinates consist of milky-white, rod-shaped (3–7 mm by 0.3–0.4 mm), composed of homogeneous, adhesive mucous within which 1–15 larvae are embedded. The conglutinates swell upon release from the hypertonic gill solution into hypotonic ambient conditions eliciting a writhing and quivering action until equilibrium is reached. Swelling forced larvae to the exterior of the conglutinate where they will gape their valves while remaining tethered to the conglutinate by a larval thread.

NA-no data for host infection strategies specific to this species.

**nHostSpecies:** Number of fish species reported to serve as larval hosts (primary or secondary) either from laboratory or natural infestations^44^ NA- no data available for the species.

**nHostGenera:** Number of fish genera reported to serve as larval hosts (primary or secondary) either from laboratory or natural infestations^44^ NA- no data available for the species.

**nHostFamily:** Number of fish families reported to serve as larval hosts (primary or secondary) either from laboratory or natural infestations^44^. NA- no data available for the species.

**mud:** Indicates associations with (true) substrate type or not (true) Mud substrate; particle size <2 mm. NA- no data available for the species.

**sand**: Indicates associations with (true) substrate type or not (false) Sand substrate; particle size <2 mm. NA- no data available for the species.

**claySilt:** Indicates associations with (true) substrate type or not (false) Clay or silt substrate; particle size <2 mm. NA- no data available for the species.

**gravel:** Indicates associations with (true) substrate type or not (false) Gravel substrate; particle size = 2–16 mm. NA- no data available for the species.

pebble: Indicates associations with (true) substrate type or not (false). Pebble substrate; particle size = 16–64 mm. NA – no data available for the species.

**cobble:** Indicates associations with (true) substrate type or not (false) Cobble substrate; particle size = 65–256 mm. NA- no data available for the species.

**boulder:** Indicates associations with (true) substrate type or not (false) Boulder substrate; particle size >256. NA- no data available for the species.

**bedrock:** Indicates associations with (true) substrate type or not (false) Bedrock substrate; smooth surface. NA- no data available for the species.

macrophyte: Indicates associatios with (true) aquatic plants or not (false). Na-no data available for the species.

**lotic:** Indicates associations with (true) habitat type or not (false). Lotic preference. NA- no data available for the species.

**lentic:** Indicates associations with (true) habitat type or not (false). Lentic preference; includes flood plain lakes and reservoirs. NA- no data available for the species.

**largeRiver:** Indicates associations with (true) habitat type or not (false). Medium to large rivers as referred in source. NA- no data available for the species.

**smallRiver:** Indicates associations with (true) habitat type or not (false). Streams to small rivers as referred in source. NA- no data available for the species.

**creek:** Indicates associations with (true) habitat type or not (false). Creek is used in the species habitat description in source. NA- no data available for the species.

**lacustrine:** Indicates associations with (true) habitat type or not (false). Preferences for natural lake systems; excludes reservoirs. NA- no data available for the species.

**slowCurrent:** Indicates associations with (true) habitat type or not (false). Slow current speed as described in source. NA- no data available for the species.

**moderateCurrent:** Indicates associations with (true) habitat type or not (false). Moderate current speed as described in source. NA- no data available for the species.

**fastCurrent:** Indicates associations with (true) habitat type or not (false). Fast current speed as described in source. NA- no data available for the species.

**larvalMinLT05:** Minimum LT05, or lethal temperature where 5% of the population will experience mortality, reported for larvae when multiple estimates of LT05 are available for that species. If no minimum is reported, that means there is only one estimate of LT05 reported for that species, which is shown in the mean. NA- no data available for the species.

**larvalMaxLT50:** Maximum reported LT05 reported for larvae when multiple estimates of LT05 are available for that species. If no max is reported, that means there is only one estimate of LT05 reported for that species, which is shown in the mean. LT05 is the lethal temperature where 5% of the population will experience mortality. NA- no data available for the species.

**larvalMeanLT05:** Mean of all reported LT05s reported for larvae when multiple estimates of LT05 are available for that species OR the single LT05 estimate that has been reported for that species. LT05 is the lethal temperature where 5% of the population will experience mortality. NA- no data available for the species.

**larvalMinLT50:** Minimum reported LT50 reported for larvae when multiple estimates of LT05 are available for that species. If no minimum is reported, that means there is only one estimate of LT05 reported for that species, which is shown in the mean. LT50 is the lethal temperature where 50% of the population will experience mortality. NA- no data available for the species.

**larvalMaxLT50:** Maximum reported LT50 reported for larvae when multiple estimates of LT05 are available for that species. If no max is reported, that means there is only one estimate of LT05 reported for that species, which is shown in the mean. LT50 is the lethal temperature where 50% of the population will experience mortality. NA- no data available for the species.

**larvalMeanLT50**: Mean of all reported LT50s reported for larvae when multiple estimates of LT05 are available for that species OR the single LT05 estimate that has been reported for that species. LT50 is the lethal temperature where 50% of the population will experience mortality. NA- no data available for the species.

**juvenileMinLT05:** Minimum reported LT05 reported for juveniles when multiple estimates of LT05 are available for that species. If no minimum is reported, that means there is only one estimate of LT05 reported for that species, which is shown in the mean. LT05 is the lethal temperature where 5% of the population will experience mortality. NA- no data available for the species.

**juvenileMaxLT05:** Maximum reported LT05 reported for juveniles when multiple estimates of LT05 are available for that species. If no max is reported, that means there is only one estimate of LT05 reported for that species, which is shown in the mean. LT05 is the lethal temperature where 5% of the population will experience mortality. NA- no data available for the species.

**juvenileMeanLT05:** Mean of all reported LT05s reported for juveniles when multiple estimates of LT05 are available for that species OR the single LT05 estimate that has been reported for that species. LT05 is the lethal temperature where 5% of the population will experience mortality. NA- no data available for the species.

**juvenileMinLT50:** Minimum reported LT50 reported for juveniles when multiple estimates of LT05 are available for that species. If no minimum is reported, that means there is only one estimate of LT05 reported for that species, which is shown in the mean. LT50 is the lethal temperature where 50% of the population will experience mortality. NA- no data available for the species.

**juvenileMaxLT50:** Maximum reported LT50 reported for juveniles when multiple estimates of LT05 are available for that species. If no max is reported, that means there is only one estimate of LT05 reported for that species, which is shown in the mean. LT50 is the lethal temperature where 50% of the population will experience mortality. NA- no data available for the species.

**juvenileMeanLT50:** Mean of all reported LT50s reported for juveniles when multiple estimates of LT05 are available for that species OR the single LT05 estimate that has been reported for that species. LT50 is the lethal temperature where 50% of the population will experience mortality. NA- no data available for the species.

**adultMinLT05:** Minimum reported LT05 reported for adults when multiple estimates of LT05 are available for that species. If no minimum is reported, that means there is only one estimate of LT05 reported for that species, which is shown in the mean. LT05 is the lethal temperature where 5% of the population will experience mortality. NA- no data available for the species.

**adultMaxLT05:** Maximum reported LT05 reported for adults when multiple estimates of LT05 are available for that species. If no max is reported, that means there is only one estimate of LT05 reported for that species, which is shown in the mean. LT05 is the lethal temperature where 5% of the population will experience mortality. NA- no data available for the species.

**adultMeanLT05:** Mean of all reported LT05s reported for adults when multiple estimates of LT05 are available for that species OR the single LT05 estimate that has been reported for that species. LT05 is the lethal temperature where 5% of the population will experience mortality. NA- no data available for the species.

**adultMinLT50:** Minimum reported LT50 reported for adults, when multiple estimates of LT05 are available for that species. If no minimum is reported, that means there is only one estimate of LT05 reported for that species, which is shown in the mean. LT50 is the lethal temperature where 50% of the population will experience mortality. NA- no data available for the species.

**adultMaxlt50:** Maximum reported LT50reported for adults when multiple estimates of LT05 are available for that species. If no max is reported, that means there is only one estimate of LT05 reported for that species, which is shown in the mean. LT50 is the lethal temperature where 50% of the population will experience mortality. NA- no data available for the species.

**adultMeanlt50:** Mean of all reported LT50s reported for adults when multiple estimates of LT05 are available for that species OR the single LT05 estimate that has been reported for that species. LT50 is the lethal temperature where 50% of the population will experience mortality.

**adultCTMax**: Mean of all reported critical thermal maximum values for adults of each species. NA- no data available for the species.

**federalStatus:** Listing under the United States of America Endangered Species Act. Categories are defined as:

endangered- any species which is in danger of extinction throughout all or a significant portion of its range.

threatened-any species which is likely to become an endangered species within the foreseeable future throughout all or a significant portion of its range.

petitioned_or_candidate- species for which the U.S. Fish and Wildlife Service (FWS) has sufficient information on their biological status and threats to propose them as endangered or threatened under the Endangered Species Act (ESA), but for which development of a proposed listing regulation is precluded by other higher priority listing activities.

not_listed- Species is not currently listedunder the Endangered Species Act (ESA). Some species may be not-listed because there has not been a petition for listing yet, which could be due to insufficient information or oversight. Some species may be not-listed because they have been delisted. Species can be delisted if the species has recovered to the point that is no longer needs protection under ESA. The original information warranting listing is demonstrated as inaccurate or new information suggests the species in not in need of protection. Extinct species can be scored “not_listed” because once they are declared extinct they are “delisted” (=not_listed) and many species went extinct prior to listing (=not_listed).

**conservationStatus:** A binary classification system that column used to account for where the USFWS designations incompletely captured decline.

listed- if its ESA Listing Status was Endangered, Threatened, or Extincted according to the USFWS Species Data Explorer (https://ecos.fws.gov/ecp/report) accessed on September 13, 2022. Many species that are widely considered to be extinct^34^ were also categorized this way.

Non-listed-species included all other ESA Listing Status categories (i.e., Proposed Endangerd, Proposed Threatened, Resolved Taxon, Species of Concern, Status Undefined, and Under Review) and all unassessed species.

**gRank:** Global Rank as defined on NatureServe. Categories are defined as:

GX–(Presumed Extinct) — Not located despite intensive searches and virtually no likelihood of rediscovery.

GH-(Possibly Extinct) Known from only historical occurrences but still some hope of rediscovery.

G1-(Critically imperiled) At very high risk of extinction or elimination due to very restricted range, very few populations or occurrences, very steep declines, very severe threats, or other factors.

G2-(Imperiled) At high risk of extinction or elimination due to restricted range, few populations or occurrences, steep declines, severe threats, or other factors.

G3-At moderate risk of extinction or elimination due to a fairly restricted range, relatively few populations or occurrences, recent and widespread declines, threats, or other factors. (Vulnerable).

G4-(Apparently secure) At fairly low risk of extinction or elimination due to an extensive range and/or many populations or occurrences, but with possible cause for some concern as a result of local recent declines, threats, or other factors.

G5-(Secure) At very low risk of extinction or elimination due to a very extensive range, abundant population or occurrences, and little to no concern from declines or threats.

GU-(Unrankable) Currently unrankable due to lack of information or due to substantially conflicting information about status or trends.

GNR-(Unranked) Global rank not yet assessed.

**mitochondrialsequences:** Indicates presence (true) or absence (false) of mitochondrial sequences on GENBANK. NA- no data of this classification available for the species.

**nuclearSequencesInclrRNAexcMSATclones**: Indicates presence (true) or absence (false) of nuclear sequences on GENBANK.

**otherGenbankDataTypes:** If additional data types were present beyond single gene sequences for a species (whole mitochondrial genome, expressed sequence tags, RNA/transcriptome sequences, whole genome) in GENBANK or REFSEQ databases these were also noted. NA- no data of this classification available for the species.

**microsatellites:** Indicated presence (true) or absences (false) of microsatellite data.

**sra:** Indicated presence or absences for high throughput sequencing data in the National Center for Biotechnology Information Sequence Read Archive; NA = no data; WGS = whole genome shotgun sequencing; RNA if RNA sequencing or transcriptome data were available; RAD = restriction site associated DNA sequencing by any method; AHE = Anchored hybrid enrichment or other hybrid capture data; Microbiome 16S = availability of 16S amplicon sequencing of bacterial community.

**noccs**: Number of georeferenced occurrences from 45 museum collections in the United States that were in the species range, were not duplicated, were geolocated within the same state listed on the lot, and were within the National Hydrography Dataset. These were used to calculate geographic trait values. NA- no data available for the species.

**nhuc8:** Number of HUC8s that contained occurrences described in noccs. NA- no data available for the species.

**aooHUC8sqkm:** Area of occupancy, measured as the total area of the HUC8s that included at least one occurrence point. NA- no data available for the species.

**totalAOOwithyear:** Area of occupancy, measured identically to above but only for records that had temporal (year) data. NA- no data available for the species.

**minLongitude:** Minimum decimal degree longitude of any occurrence of the species. NA- no data available for the species.

**minLatitude:** Minimum decimal degree latitude of any occurrence of the species. NA- no data available for the species.

**maxLongitude:** Maximum decimal degree longitude of any occurrence of the species. NA- no data available for the species.

**maxLatitude:** Maximum decimal degree latitude of any occurrence of the species. NA- no data available for the species.

**midLongitude:** Decimal degree longitude of the center of the convex polygon that includes all occurrences of the species in Albers projection. NA- no data available for the species.

**midLatitude:** Decimal degree latitude of the center of the convex polygon that includes all occurrences of the species in Albers projection. NA- no data available for the species.

**modeStreamOrder:** Mode Strahler stream order of occurrences of the species that were snapped to NHDPlusV2 flowlines with stream order >1. NA- no data available for the species.

**iqrStreamOrder:** Interquartile range of Strahler stream order of occurrences of the species that were snapped to NHDPlusV2 flowlines with stream order >1. NA- no data available for the species.

**medianStreamSlope**: Median stream slope (slope of flowline (meters/meters) based on smoothed elevations) of occurrences of the species that were snapped to NHDPlusV2 flowlines with stream order >1. NA- no data available for the species.

**iqrStreamSlope:** Interquartile range of stream slope (slope of flowline (meters/meters) based on smoothed elevations) of occurrences of the species that were snapped to NHDPlusV2 flowlines with stream order >1. NA- no data available for the species.

**medianQAMA:** Median of mean annual discharge (flow from runoff (cubic feet per second)) of occurrences of the species that were snapped to NHDPlusV2 flowlines with stream order >1. See NHDPlusV2 documents^61^ for definition of QAMA. NA- no data available for the species.

**iqrQAMA:** Interquartile range of mean annual discharge (flow from runoff (cubic feet per second)) of occurrences of the species that were snapped to NHDPlusV2 flowlines with stream order >1. See NHDPlusV2 documents^61^ for definition of QAMA. NA- no data available for the species.

**medianVAMA:** Median annual velocity (for QAMA (feet per second)) of occurrences of the species that were snapped to NHDPlusV2 flowlines with stream order >1. See NHDPlusV2 documents for definition of VAMA. NA- no data available for the species.

**iqrVAMA:** Interquartile range of the mean annual velocity (for QAMA (feet per second)) of occurrences of the species that were snapped to NHDPlusV2 flowlines with stream order >1. See NHDPlusV2 documents for definition of VAMA. NA- no data available for the species.

**nStreamCharacteristicLots:** Number of occurrences used to estimate the stream characteristic traits. Only occurrences within the NHDPlusV2 dataset (which does not include Alaska) and with non-negative flow velocity, discharge, and stream slope were used and therefore this value may be less than noccs. NA- no data available for the species.

**mississippiEmbayment:** We considered a species present (present) in this region if it had >2 occurrences, questionable if 1–2 occurrences (questionable), and absent if no occurrences (absent). All biogeographic definitions follow Haag (2009)^53^. Encompasses the lower Mississippi River and all tributaries below the mouth of the Ohio River, including most the Lower Red River system and the Atchafalaya basin, and the Mermentau, with flow into the Gulf of Mexico just east of the Atchafalaya basin.

**upperMississippi:** We considered a species present (present) in this region if it had >2 occurrences, questionable if 1–2 occurrences (questionable), and absent if no occurrences (absent). Includes the entire Mississippi River system upstream of the Ohio River, excluding the Missouri River system except for the southern tributaries of the lower Missouri River.

**ohioan:** We considered a species present (present) in this region if it had >2 occurrences, questionable if 1–2 occurrences (questionable), and absent if no occurrences (absent). Includes the Ohio River and all of its tributaries except the upper two-thirds of the Cumberland and Tennessee River systems.

**tennesseeCumberland:** We considered a species present (present) in this region if it had >2 occurrences, questionable if 1–2 occurrences (questionable), and absent if no occurrences (absent). Includes the upper two-thirds of the Tennessee and Cumberland River systems. The downstream boundary of the provinces is located downstream of Muscle Shoals in the Tennessee River in northwestern AL and in the Cumberland River near Clarksville, TN. Boundaries are set based on the occurrences of endemic species.

**interiorHighlands**: We considered a species present (present) in this region if it had >2 occurrences, questionable if 1–2 occurrences (questionable), and absent if no occurrences (absent). Encompasses two geographically discontinuous regions: 1.) upper White and upper St. Francis systems with the Ozark Plateaus province plus the adjacent Verdigris, Neosho, and Illinois River systems. 2) The upper Ouachita, Kiamichi, and Little river systems, and the Poteau River.

**greatPlains:** We considered a species present (present) in this region if it had >2 occurrences, questionable if 1–2 occurrences (questionable), and absent if no occurrences (absent). Includes all river systems east of the Rocky Mountains, from the upper Red River of Texas and Oklahoma, north to the Nelson-Churchill basins in central Canada.

**stLawrenceGreatLakes:** We considered a species present (present) in this region if it had >2 occurrences, questionable if 1–2 occurrences (questionable), and absent if no occurrences (absent). Includes all five Great Lakes and Lake St. Clair and their watersheds. Also includes St. Lawrence and Ottawa river systems and rivers flowing into James Bay.

**westernGulf:** We considered a species present (present) in this region if it had >2 occurrences, questionable if 1–2 occurrences (questionable), and absent if no occurrences (absent). All rivers flowing into the Gulf of Mexico from the Brazos south to the Rio Grande.

**sabineTrinity:** We considered a species present (present) in this region if it had >2 occurrences, questionable if 1–2 occurrences (questionable), and absent if no occurrences (absent). Encompasses rivers of the central Gulf Coast.

**pontchartrainPearlPascagoula:** We considered a species present (present) in this region if it had >2 occurrences, questionable if 1–2 occurrences (questionable), and absent if no occurrences (absent). Includes the Pearl and Pascagoula River systems and all streams that flow into Lakes Pontchartrain and Maurepas.

**mobileBasin:** We considered a species present (present) in this region if it had >2 occurrences, questionable if 1–2 occurrences (questionable), and absent if no occurrences (absent). All rivers flowing into Mobile Bay in the Gulf of Mexico.

**escambiaChoctawhatchee:** We considered a species present (present) in this region if it had >2 occurrences, questionable if 1–2 occurrences (questionable), and absent if no occurrences (absent). Includes the Escambia, Yellow, and Choctawhatchee rivers.

**apalachicolan:** We considered a species present (present) in this region if it had >2 occurrences, questionable if 1–2 occurrences (questionable), and absent if no occurrences (absent). Includes the Apalachicola, Ochlockonee, and Suwannee rivers, and Econfina Creek that flows into the Gulf of Mexico.

**peninsularFlorida:** We considered a species present (present) in this region if it had >2 occurrences, questionable if 1–2 occurrences (questionable), and absent if no occurrences (absent). Extends from Waccasassa River on the Florida Gulf Coast, south and around the Florida Peninsula, north to and including St. Mary’s River.

**southernAtlantic**: We considered a species present (present) in this region if it had >2 occurrences, questionable if 1–2 occurrences (questionable), and absent if no occurrences (absent). Satilla River north to the James River.

**northernAtlantic:** We considered a species present (present) in this region if it had >2 occurrences, questionable if 1–2 occurrences (questionable), and absent if no occurrences (absent). Extends from the York River system north of Chesapeake Bay to Newfoundland.

**pacific:** We considered a species present (present) in this region if it had >2 occurrences, questionable if 1–2 occurrences (questionable), and absent if no occurrences (absent). All rivers in the United States of America flowing into the Pacific Ocean; Headwaters of the of the upper Missouri River above Great Falls.

### Second file

**Identity:** SHELDmusselFishData2023AUG.csv

**Size:** 152.4KB

**Format and storage mode:** CSV

**Contents:** Long format data set with known fish hosts for each mussel species. The first column is the mussel scientificName and the last three columns (A-C) are hierarchical Linnaean classifications for fish host families, genera, and species with each row representing a fish host-mussel association.

**Variable information:**

**musselScientificName:** Scientific name used under the Linnaean classification system^41,57,58^.

**fishFamily:** A list of the family used under the Linnaean classification system of fishes known to serve as larval hosts (primary or secondary) either from laboratory or natural transformations. Names are current with the American Fisheries Society^53^.

**fishGenus:** A list of the genera used under the Linnaean classification system of fishes known to serve as larval hosts (primary or secondary) either from laboratory or natural transformations. Names are current with the American Fisheries Society^53^.

**fishSpecies:** A list of the specific epithet used under the Linnaean classification system of fishes known to serve as larval hosts (primary or secondary) either from laboratory or natural transformations. Names are current with the American Fisheries Society^53^.

### Third file

**Identity:** SHELDgrowthRates2023AUG

**Size:** 31.6 KB

**Format and storage mode:** CSV

**Contents:** Contains growth rates for mussel species found in the United States of America. Each row represents a population of a species such that one species may have several rows if growth rates were estimated for several populations.

**Variable information:**

**scientificName:** Scientific name used under the Linnaean classification system ^41,53,57^.

**growthRate:** K value estimated from the von Bertalanffy equation by the original source

**sampleSize:** number of individuals in the data set.

**sexIdentified:** the sex of the animals used in the study (male or female).

**methodUsed:** The method used to estimate growth rate. Typically, vonBertalanffy equations were used. There is one case where absolute growth rate was measured.

**location:** the geographic location the animals were collected from in the United States of America as reported by the original source. We provide a specific location and the two-letter abbreviation for the state separated by a comma.

**reference:** A list of references the data for each growth rate was sourced from.

### Fourth file

**Identity:** SHELDreferenceList2023AUG.xlsx

**Size:** 359.2 KB

**Format and storage mode:** xlsx

**Contents:** A complete list of mussel scientificNameAuthority with references for traitName found in columns J to BS in SHELDspeciesTraitMatrix2023AUG.xlsx

**Variable information:**

**traitName:** references to the traits in the complete dataset SHELDspeciesTraitMatrix2023AUG.xlsx. The trait levels included here are those we collated data for from the search. Traits are listed below with a brief definition. Complete definitions for each trait and trait subcategory can be found above in the variable information for the first file.

**tsn:** Taxonomic Serial Number from Integrated Taxonomic Information System. Represent a persistent identifier to facilitate tracing name changes in the future. May not reflect all name changes accepted by FMCS 2021. NA- no data of this classification available for the species.

**natureServeElement:** Unique identifier for NatureServe queries.

**meanLength:** Mean of shell lengths. Measured across the anterior-posterior plane of the shell.

**maxLength**: Maximum shell length (length description) in millimeters. Some records may estimate asymptotic length. We assumed that the difference between maximum observed length and asymptotic length is negligible. Measured across the anterior-posterior plane of the shell.

**shellSculpture:** Species are considered sculptured if the shell exhibits knobs, pustules, spines, corrugations, or undulations. This binary classification was based on photographs of material from museum lots found on MolluscaBase^42^, Musselp^43^, and species accounts^46,47,59^.

**larvalDescription:** Descriptions that reflect two discrete larval characteristics. The term “hooked” is applied indiscriminately to independently derived structures that function similarly, to grab onto the host fish. Hookless refers to the absence of such a structure. Here, the larvae are hooked or hookless combined with terms describing their shape based on Hoggarth^49^.

**larvalHeight:** Mean height of larvae in micrometers (µm) measured perpendicular to the hinge. Max height is given if no mean is reported.

**larvalLength:** Mean length of larvae in micrometers (µm) measured parallel to the hinge.

**matureAge:** Mean, median, or modal age at maturity in years for females. Where different ages at maturity were gathered for distinct populations, the best supported records were averaged. Species that are known to mature prior to age one are defined as 0. Male maturity age was accepted where female data were not available. NA- no data of this classification available for the species.

**maxAge:** Longevity in years based on life in the wild wherever available. Where not indicated, the record was assumed to be from the wild. If wild records were not known, records for captive individuals were considered.

**growthRate:** Binary indicator of the availability of growth rate data for each species. Literature sources often evaluated growth rates inconsistently. We dealt with this by including a separate sheet of growth rates, SHELDgrowthRates2023AUG.csv. That sheet details growth rates, sample sizes, location of the study, and the data source.

**fecundity:** Refers to mean number of eggs produced by a single female in one brood. There were not studies strictly evaluating the production of multiple clutches per year.

**brood:** long_term indicates long (bradytictic)- or short_term indicates short term (tachytictic) brooding strategy; long term brooders hold larvae from fall to spring or summer, whereas short term brooders hold larvae from winter to spring or summer. NA- no data of this classification available for the species.

**marsupialGills:** Indicates that brooding of larvae occurs in outer gills only (ectobranchy) or all four gills (tetrageny).

**hermaphrodite:** Indicates reports of hermaphroditic populations.

**hostInfectionStrategy:** Category of primary host infection strategy. Categories are defined above in the variable information for the first file.

**nHostSpecies:** Number fish species reported to serve as larval hosts (primary or secondary) either from laboratory or natural infestations^44^.

**nHostGenera**: Number fish genera reported to serve as larval hosts (primary or secondary) either from laboratory or natural infestations^44^.

**nHostFamily:** Number of fish families reported to serve as larval hosts (primary or secondary) either from laboratory or natural infestations^44^.

**habitat:** Combines the trait sub-categories in SHELDspeciesTraitMatrix2023AUG.xlsx. describing species associations with substrate type, lentic and lotic systems collated from the literature. Trait subcategories include sand, claySilt, gravel, cobble, boulder, bedrock, lotic, lentic, largeRiver, smallRiver, creek, lacustrine, slowCurrent, moderateCurrent, and fastCurrent. These subcategories are defined above in the variable information for the first file.

**larvalMinLT05:** Minimum LT05, or lethal temperature where 5% of the population will experience mortality, reported for larvae when multiple estimates of LT05 are available for that species. If no minimum is reported, that means there is only one estimate of LT05 reported for that species, which is shown in the mean. NA- no data available for the species.

**larvalMaxLT50:** Maximum reported LT05 reported for larvae when multiple estimates of LT05 are available for that species. If no max is reported, that means there is only one estimate of LT05 reported for that species, which is shown in the mean. LT05 is the lethal temperature where 5% of the population will experience mortality. NA- no data available for the species.

**larvalMeanLT05:** Mean of all reported LT05s reported for larvae when multiple estimates of LT05 are available for that species OR the single LT05 estimate that has been reported for that species. LT05 is the lethal temperature where 5% of the population will experience mortality. NA- no data available for the species.

**larvalMinLT50:** Minimum reported LT50 reported for larvae when multiple estimates of LT05 are available for that species. If no minimum is reported, that means there is only one estimate of LT05 reported for that species, which is shown in the mean. LT50 is the lethal temperature where 50% of the population will experience mortality. NA- no data available for the species.

**larvalMaxLT50:** Maximum reported LT50 reported for larvae when multiple estimates of LT05 are available for that species. If no max is reported, that means there is only one estimate of LT05 reported for that species, which is shown in the mean. LT50 is the lethal temperature where 50% of the population will experience mortality. NA- no data available for the species.

**larvalMeanLT50**: Mean of all reported LT50s reported for larvae when multiple estimates of LT05 are available for that species OR the single LT05 estimate that has been reported for that species. LT50 is the lethal temperature where 50% of the population will experience mortality. NA- no data available for the species.

**juvenileMinLT05:** Minimum reported LT05 reported for juveniles when multiple estimates of LT05 are available for that species. If no minimum is reported, that means there is only one estimate of LT05 reported for that species, which is shown in the mean. LT05 is the lethal temperature where 5% of the population will experience mortality. NA- no data available for the species.

**juvenileMaxLT05:** Maximum reported LT05 reported for juveniles when multiple estimates of LT05 are available for that species. If no max is reported, that means there is only one estimate of LT05 reported for that species, which is shown in the mean. LT05 is the lethal temperature where 5% of the population will experience mortality. NA- no data available for the species.

**juvenileMeanLT05:** Mean of all reported LT05s reported for juveniles when multiple estimates of LT05 are available for that species OR the single LT05 estimate that has been reported for that species. LT05 is the lethal temperature where 5% of the population will experience mortality. NA- no data available for the species.

**juvenileMinLT50:** Minimum reported LT50 reported for juveniles when multiple estimates of LT05 are available for that species. If no minimum is reported, that means there is only one estimate of LT05 reported for that species, which is shown in the mean. LT50 is the lethal temperature where 50% of the population will experience mortality. NA- no data available for the species.

**juvenileMaxLT50:** Maximum reported LT50 reported for juveniles when multiple estimates of LT05 are available for that species. If no max is reported, that means there is only one estimate of LT05 reported for that species, which is shown in the mean. LT50 is the lethal temperature where 50% of the population will experience mortality. NA- no data available for the species.

**juvenileMeanLT50:** Mean of all reported LT50s reported for juveniles when multiple estimates of LT05 are available for that species OR the single LT05 estimate that has been reported for that species. LT50 is the lethal temperature where 50% of the population will experience mortality. NA- no data available for the species.

**adultMinLT05:** Minimum reported LT05 reported for adults when multiple estimates of LT05 are available for that species. If no minimum is reported, that means there is only one estimate of LT05 reported for that species, which is shown in the mean. LT05 is the lethal temperature where 5% of the population will experience mortality. NA- no data available for the species.

**adultMaxLT05:** Maximum reported LT05 reported for adults when multiple estimates of LT05 are available for that species. If no max is reported, that means there is only one estimate of LT05 reported for that species, which is shown in the mean. LT05 is the lethal temperature where 5% of the population will experience mortality. NA- no data available for the species.

**adultMeanLT05:** Mean of all reported LT05s reported for adults when multiple estimates of LT05 are available for that species OR the single LT05 estimate that has been reported for that species. LT05 is the lethal temperature where 5% of the population will experience mortality. NA- no data available for the species.

**adultMinLT50:** Minimum reported LT50 reported for adults, when multiple estimates of LT05 are available for that species. If no minimum is reported, that means there is only one estimate of LT05 reported for that species, which is shown in the mean. LT50 is the lethal temperature where 50% of the population will experience mortality. NA- no data available for the species.

**adultMaxLT50:** Maximum reported LT50reported for adults when multiple estimates of LT05 are available for that species. If no max is reported, that means there is only one estimate of LT05 reported for that species, which is shown in the mean. LT50 is the lethal temperature where 50% of the population will experience mortality. NA- no data available for the species.

**adultMeanLT50:** Mean of all reported LT50s reported for adults when multiple estimates of LT05 are available for that species OR the single LT05 estimate that has been reported for that species. LT50 is the lethal temperature where 50% of the population will experience mortality.

**adulCTMax**: Mean of all reported critical thermal maximum values for adults of each species. NA- no data available for the species.

**federalStatus:** Listing under the United States of America Endangered Species Act.

**conservationStatus:** A binary classification system that column used to account for where the USFWS designations may incompletely captured decline.

**gRank:** Global Rank as defined on NatureServe.

**mitochondrialSequences:** Indicates presence (YES) or absence (NO)of mitochondrial sequences on GENBANK. NA- no data of this classification available for the species.

**nuclearSequencesInclrRNAexcMSATclones**: Indicates presence (YES) or absence (NO) of nuclear sequences on GENBANK.

**otherGenbankDataTypes:** If additional data types were present beyond single gene sequences for a species (whole mitochondrial genome, expressed sequence tags, RNA/transcriptome sequences, whole genome) in GENBANK or REFSEQ databases these were also noted NA- no data of this classification available for the species.

**microsatellites:** Indicated presence (YES) or absences (NO) of microsatellite data.

**sra:** Indicated presence or absences for high throughput sequencing data in the National Center for Biotechnology Information Sequence Read Archive.

**referenceID1:** Citation or museum catalogue number for the traitName identified. NA - no data available for the species. To derive traitValues for some traitNames, it was necessary to use multiple sources. In such cases, an additional column or two will follow and will be idenfitied as **referenceID2** or **referenceID3**.

**referenceID2:** Citation or museum catalogue number for the traitName identified. NA - no data available for the species.

**referenceID3:** Citation or museum catalogue number for the traitName identified. NA - no data available for the species.

### Fifth file

**Identity:** SHELDspeciesStateMatrix.csv

**Size:** 37.0 KB

**Format and storage:** CSV

**Contents:** Association matrix of political boundaries for states in the United States of America and each mussel species. The first column is the name of the state political boundary. The remaining column headers (B-KP) correspond to the scientificName column SHELDspeciesTraitMatrix2023AUG.xlsx listed as the First file. The number one (1) is used to identify the occurrence of each species within the political boundary as indicated by NatureServe.

**Variable information:**

**state:** the name of each state political boundary in the United States of America.

## Technical Validation

Initial quality assurance and quality control procedures included having two separate authors examine data for each reference prior to including data in the complete dataset SHELDspeciesTraitMatrix2023AUG.xlsx, the host data set SHELDfishMusselMatrix2023AUG.csv, SHELDgrowthRates2023AUG.csv, and SHELDspeciesStateMatrix2023AUG.csv. Upon completion of data compilation, we checked each file for formatting errors. We checked that all text columns contained only relevant text. We checked that all numerical trait columns contained only correctly formatted numeric data input in correct units or NA when appropriate. Next, we completed an outlier analysis of numerical traits to identify potential errors for the complete dataset SHELDspeciesTraitMatrix2023AUG.xlsx. We performed three levels of outlier analysis for every numerical trait: 1) outliers among all species, 2) outliers among members of the same genus, 3) outliers among members of each phylogenetic tribe. We flagged data points as outliers if they were greater than 2.5 standard deviations of the mean of the given trait. If we flagged a data point, we rechecked the original reference. If the data point matched the value in the original reference, we made no change. If the data point did not match the value in the original reference, we either updated it to the correct value or deleted it if the value did not exist in that reference. Overall, 1.7% of the raw continuous data were flagged as outliers across all species, 4.9% were flagged as outliers at the genus level, and 2.2% were flagged as outliers at the tribe level. Of the identified outliers, 6.40% were ultimately corrected or deleted.

## Data Availability

R code and associated shapefiles for biogeographic determinations can be found at https://github.com/seanmkeogh/SHELD_biogeo.
